# Inapplicability of advance directives in a paternalistic setting: the case of a post-communist health system

**DOI:** 10.1186/1472-6939-12-12

**Published:** 2011-06-15

**Authors:** Gentian Vyshka, Jera Kruja

**Affiliations:** 1 Biomedical & Experimental Department, Faculty of Medicine, Rr. Dibrës 371, Tirana, Albania; 2 Service of Neurology, University Hospital Centre 'Mother Theresa', Rr. Dibrës 371, Tirana, Albania

## Abstract

**Background:**

The Albanian medical system and Albanian health legislation have adopted a paternalistic position with regard to individual decision making. This reflects the practices of a not-so-remote past when state-run facilities and a totalitarian philosophy of medical care were politically imposed. Because of this history, advance directives concerning treatment refusal and do-not-resuscitate decisions are still extremely uncommon in Albania. Medical teams cannot abstain from intervening even when the patient explicitly and repeatedly solicits therapeutic abstinence. The Albanian law on health care has no provisions regarding limits or withdrawal of treatment. This restricts the individual's healthcare choices.

**Discussion:**

The question of *'medically futile' *interventions and pointless life-prolonging treatment has been discussed by several authors. Dutch physicians call such interventions '*medisch zinloos*' (*senseless*), and the Netherlands, as one of the first states to legislate on end-of-life situations, actually regulates such issues through appropriate laws. In contrast, leaving an 'advance directive' is not a viable option for Albanian ailing individuals of advanced age. Verbal requests are provided during periods of mental competence, but unfortunately such instructions are rarely taken seriously, and none of them has ever been upheld in a legal or other official forum.

**Summary:**

End-of-life decisions, treatment refusal and do-not-resuscitate policies are hazardous options in Albania, from the legal point of view. Complying with them involves significant risk on the part of the physician. Culturally, the application of such instructions is influenced from a mixture of religious beliefs, death coping-behaviors and an immense confusion concerning the role of proxies as decision-makers. Nevertheless, Albanian tradition is familiar with the notion of '*amanet*', a sort of living will that mainly deals the property and inheritance issues. Such living wills, verbally transmitted, may in certain cases include advance directives regarding end-of-life decisions of the patient including refusal or termination of futile medical treatments. Since these living wills are never formally and legally validated, their application is impossible and treatment refusal remains still non practicable. Tricks to avoid institutional treatment under desperate conditions are used, aiming to provide legal coverage for medical teams and relatives that in extreme situations comply with the advice of withholding senseless treatment.

## Background

Until a few decades ago the application of a paternalistic philosophy in the state-run medical health system was normal practice in Albania. Decisions by the medical staff were rarely, if ever, discussed with patients. According to this philosophy, the state cared for everybody and for everything, even the details of everyday life. The medical system was a part of this state mechanism, and therefore all decisions of medical teams were not subject to question. The paternalistic and communist society, and the totalitarian system, created a reality in which even the individual's ultimate fate was a matter for society to determine [1].

On the other hand, not providing medical aid or resuscitation to an ailing person seems out of the question to [virtually] all Albanian practitioners, and even to paramedicals and laymen. After all, isn't that what medical institutions exist for? With the exclusion of clear and unequivocal signs of death (rigor mortis etc.), other situations need to be treated more or less aggressively by inserting venous lines, defibrillating, massaging the heart externally, ventilating the patient artificially, intubating him, delivering infusions and drugs intravenously or even intracardially etc. All these procedures should make medical sense; overtreating will obviously not help dying people to escape death. But can people explicitly refuse or disapprove procedures? *'Do not intubate me' *is a frequent request in other cultures [2]. Senseless interventions will deliver false expectations to the patient and to his/her relatives, while frustrating the supportive staff. Moreover, the fact that an intervention is senseless will raise doubts about the professional judgment of the treating clinician himself, apart from wasting public resources. This position might also exacerbate the scarcity of means at the disposal of Albanian public medical facilities.

The anecdotal vignette below illustrates a situation that occurs frequently in Albanian medical facilities. Note, however, that it is a somewhat exaggerated depiction of what is frequently present in real life cases, maybe in less dramatic form.

*94 year old Caucasian male is brought in a hospital facility after three and half hours of bumpy driving on a very rough road. He has been suffering from hypertension and atrial fibrillation for more than three decades. He has smoked 20 cigarettes per day for forty years and had a strong ethanol addiction until fourteen years ago when the diagnosis of a diffuse hematemesis and an erosive gastritis forced him to stop consuming alcohol. Upon arrival he is in deep stupor (Glasgow Coma Scale rating eight to nine points) with a left hemiplegic deficit and febrile (39°C). No signs of meningeal irritation were detected*.

*The paramedics accompanying the patient witnessed the arguing of relatives about the issue of sending or not the old man to hospital. In fact, days before, the old man had declared to his wife (87 years of age) that he wanted to 'die at home'. But his wife, mother of seven sons and two daughters, was excluded from the decision-making since she was considered from the eldest son as 'extremely emotional', and even was denied to accompany her husband in the hospital*.

*On the way to the hospital the old man was accompanied by three of his sons and one of the daughters; the latter came back home recently after a period living abroad. The continuous weeping of the daughter, who had been away from the paternal home for several years, forced the emergency practitioner to immediately hospitalize the 94 years old man*.

*The man was intubated and received infusions, antibiotics and antihypertensives. During the third day of hospitalization the patient died, after a bilateral areactive mydriasis proved the irreversibility of the cerebral damage. However, even during the last twenty-four hours of his life, the relatives kept on requiring medical treatment, until death was pronounced, despite the fact that the medical staff had explained the hopelessness of the situation several times*.

This medical scenario illustrates fairly well how Albanian culture, and probably other European cultures as well, are death-denying cultures [3]. Albanian epos and traditional beliefs have even considered death as a divine curse [4]. There are differences, of course, between a death-denying culture and the inability of people to accept the inevitable death of a loved one. Although the death-denying character of the Albanian setting is clear, however, the parental desires are generally respected from the descendants, provided the desires have been openly and clearly formulated during periods of mental competence. Such a notion of verbal desire condensed in the form of an 'amanet' (an Albanian term for a living will; see below) was in fact communicated to the wife of the old man; but since nobody else witnessed or took care of validating it, the option of 'dying at home' was ignored, and the case became an example of futile treatment.

Considering the above illustrative case, sometimes things can be viewed conversely; i.e. a patient is supposed that he/she did not gave in advance the approval for hospitalization, and this will not be considered as a 'refusal', but rather elusively will be equivalent with a request for continuing an outpatient treatment (at home); although such a treatment will be largely inexistent, as it might be a home treatment for severely ill and dying patients. In fact, disguising an indirect refusal (which is forbidden in its open and straightforward form) as a 'missed approval' is a possible way out, together with the option of 'home-based treatment'; both of which effectively in desperate cases are very similar to a treatment withdrawal or refusal.

Albanian medical teams are obviously hesitant to abstain from emergency hospitalizations and even more, are not in the position to comply with end-of-life decisions. This hesitation is proportionally related to senseless hospitalizations and with unnecessary interventions (invasive and non-invasive). However, it is not a willing hesitation. Albanian law on health care leaves no option to deny treatment, and absolutely no provisions are foreseen in cases when a DNR or treatment withdrawal request is made [5]. The law merely provides an article regarding the 'approval**' **of treatment requested by the patient (6.2.c; *ibid*); the option of treatment refusal is not foreseen.

The discussion of end-of-life issues seems more elaborated in countries neighboring Albania. For example, Kosovo has recently promulgated a law on the rights and responsibilities of citizens requiring health care, with detailed articles explicitly regulating the issues of treatment refusal, and of personal or proxy decisions [6]. In a study performed in Greece, 'medical paternalism' was considered as the main regulating factor in end-of-life decisions [7]. The authors of this study stated that withholding CPR (*cardiopulmonary resuscitation*) was the main operative modality in this setting.

## Discussion

Dealing with end-of-life situations, and achieving a decision in severe and hopeless medical situations, is probably one of the biggest ethical issues affecting patients and their relatives, as well as the medical staff. Life-sustaining procedures may be complicated and they obviously require effort on the part of the professionals. Volume resuscitation, defibrillation, intubation and directed ventilation are miraculous interventions, but only in well-defined medical and surgical conditions. Such procedures cannot and should not be conceived of as means of prolonging suffering and at the same time fomenting unjustified expectations among the relatives of the patient, and hence creating a surplus of inhumane suffering.

The question of *'medically futile' *interventions and excessive life-prolonging treatment has been raised by several authors. Dutch physicians define the situation '*medisch zinloos*' [*senseless*], and the Netherlands was the first state to legislate on end-of-life situations, thus regulating the issue [8]. For some authors, the notion of treatment refusal should expand to additional practical issues and to cover non-professional staff, such as the case of paramedicals facing the refusal of a patient to be hospitalized [9]. In 2006 Austria promulgated a law regulating the issue of advance directives, mainly regarding the prolongation of the treatment, albeit the Dutch experience preceded the others' [10]. Japanese scholars have elaborated the notion of *'physiological futility' *of a medical treatment, connoting the irreversibility of an already advanced pathological state. They propose that decisions on treatment withdrawal should remain shared ones [11]. The question of *medically futile *interventions has even been extended in other settings. Not only gerontologists, but neonatologists as well have expressed opinions regarding the withdrawal of artificial ventilation in the case of dying babies [12].

DNR decisions are generally included and discussed in the field of end-of-life practices. These practices are labeled differently, and it seems difficult to find a unique description of the matter. Even medical professionals describe the same applicable practice through a variety of terms and perspectives. Thus, end-of-life decisions are labeled, in the professional literature, as: a) *euthanasia*; b) *ending of life*; c) *palliative or terminal sedation*; d) *symptom alleviation *and e) *other *perspectives [13]. The conceptualization of DNR is gradually evolving; the old term is being replaced by DNAR (*do-not-attempt-resuscitation*) or even 'Allow Natural Death' [10].

The pros and cons of treatment withdrawal, or abstaining from resuscitation procedures (DNR), represent a long-standing medical and philosophical controversy. The confrontation with impending death and near-death conditions invokes a mixture of survival mechanisms that a free will cannot easily overcome (provided that free will does exist) [14]. It seems that confronting this dilemma could have been a factor at the origin of religion. Several authors have underlined the fact that the death of a relative, as an archaic feeling, may have predisposed societies to religion [15]. Under such circumstances, emotions related to the death of a relative will complicate the decision-making process, since these decisions cannot be made only from a rigid medical perspective.

Making an advance directive is one of the options for facing near-death situations, and probably a sophisticated way to cope with the death as a notion. There are different ways of giving an advance directive, such as Miller has pointed out [16]:

a. Healthcare proxy (appointing a person to decide);

b. Living will (giving in advance, specific instruction about possible future treatments);

c. DNR (advance decision about cardiopulmonary resuscitation);

d. MOLST (medical orders for life-sustaining treatment).

In Albania, leaving an 'advance directive' for sick old individuals and for octogenarians is still a non-viable option. Verbal desires and requests are sometimes expressed during periods of mental competence, but unfortunately few of them are taken seriously, and none of them up to now has been validated in a written form (i.e. through a notary act, a court decision etc.). Albanian tradition is familiar with the notion of '**amanet**', a word derived from the Turkish '**aman**', whose meaning is both 'please' and 'for goodness sake'. 'Amanete - amanetet' (plural forms of the term 'amanet', literally a supplication for God's sake), have become a synonym of living wills, although they concern also property and inheritance issues, as well as advices for the future. In some case someone dying will leave an 'amanet' to another person to take care after his children, if these are minors. Even the precise place where the dying person wants to be buried, and the funeral ceremony, might become part of an 'amanet'.

The etymology of the word, widely used in everyday Albanian vocabulary, tells a lot upon the feelings of empathy and of mercy that the supplicating person is trying to evoke with the interlocutor. Expansion of the scope of 'amanete' could be a way towards institutionalizing the concept of the living will and of advance directives in Albania; of course appropriate legal measures are needed to uphold an 'amanet' as legally binding.

## Summary

The ambivalence and the inability of the relatives to act as proxies in the process of decision-making, and the lack of legal vehicles, are becoming deleterious factors for Albanian emergency and ICU teams. A decision is even more difficult to achieve in long-term care facilities. Nevertheless, the need for a no-CPR policy has been underlined elsewhere for decades [17]. The authors insist that when a DNR policy exists, physicians and other staff are more likely to address these important issues with patients and families.

Among the tricks used to avoid forgoing institutional treatment under desperate conditions, is the formula of 'continuing the medical treatment as an outpatient case', or treating the patient 'at home'. This option is mainly related to a last-minute discharge of the patient in a near-death condition, which is a silent deal between relatives and medical staff. In this way the relatives, apart from ending a senseless hospitalization, avoid making requests for a treatment refusal, faking a following of the treatment in an ambulatory form or in an outpatient basis; hospitals on their side will declare smaller figures of cases with lethal outcome.

Long-term care facilities and hospices are a rarity in Albania, and facing the burden of treating third-age persons or chronic patients who suffer from irreversible, degenerative and strongly disabling conditions is still mainly a duty of general hospitals, or ordinary clinics. This forced role will obviously divert the means at disposal from being used in more fruitful interventions, something unacceptable for an underfunded health system.

Hesitating family members who are unable to act as proxies and to make a decision (for example, through respecting the advance directive or 'amanet' of the patient) aggravate delicate situations such as near-death ones. The situation is even worse when relatives hold ambivalent positions or contradictory ones (i.e. when one relative of the patient demands treatment withdrawal and a second one insists on the continuation of the treatment). Relatives might tend toward overtreatment or undertreatment; it is often a difficult task to find out which position would have been adopted by the patient himself in the case the latter had still some degree of mental competence. Authors suggest, for example, that Caucasian proxies commit overtreatment errors, whereas African American proxies commit undertreatment errors; both extremes can be moderated by advance planning [18].

Actually, a living will in Albanian medical settings may be an inapplicable option, culturally, religiously and legally. Culturally the death-denying position is a prevalent one, but medical ignorance might play an important role as well; in an article describing the situation of an Albanian hospice facility, it is stated that 'only but a few of the patients were aware of the near-death situation and the irreversibility of their medical condition' [19]. This is exacerbated by the uncertainty and the confusion that often surrounds near-death situations, such as those regarding the irreversibility of certain medical conditions.

We interviewed the relatives of 57 recently admitted third-age persons who had been admitted to a neurological facility due to the sudden onset of a comatose situation of different etiologies. More than 95% of the relatives had no idea at all regarding advance desires or directives expressed by the patient prior to the onset of coma. Two relatives who were aware of the reluctance of the old-age patient to be hospitalized nonetheless opted differently under the pressure of other persons. In another study, end-of-life decisions in an Albanian outpatient setting were also not uniformly perceived. For example 27% of the relatives opted for a prolongation of the medical treatment, even if the suffering of the patient was unbearable [20]. False expectations and the lack of information upon the irreversibility of the medical condition are among the possible causes pushing relatives to prolong treatment, in a setting where the medical staff is practically without legal options even to suggest any alternatives.

In the legal field, the number of malpractice suits brought to court in Albania is constantly increasing, and not only here. Authors have pointed out that frequently a medical error is inaccurately considered as a synonym of medical negligence [21]. Under such a pressure, medical staffs will obviously avoid immediate (although logical) decisions, and will unnecessarily hospitalize 'medically senseless' cases (to adopt the Dutch terminology). More and more widely the DOA (death-upon-arrival) record is being replaced by *ad hoc *medical histories intended to somehow justify the admission of patients in a desperate and irreversible condition to ICUs (Intensive Care Units). The staff of these units ultimately has their say upon stopping the treatment and declaring the death. This inappropriate use of intensive care has been considered (a) *unnecessary*, (b) *unsuccessful*, (c) *unsafe*, (d) *unkind *and (e) *unwise*, in a paper summarizing the economic considerations of ICU treatment for hopeless cases. The paper also noted the inhuman nature of false hopes created for the relatives of the patient [22].

This sad itinerary is becoming a practice in Albania, and there is justified fear that the general reticence toward making end-of-life decisions will worsen with time. There are, however, Albanian judges and judicial authors who are considering a revision of perspectives. In a recently published paper a district judge wrote that 'a physician may deliver drugs to a patient with the intent of alleviating pain even though he is aware that such an act will shorten the patient's life span' [23].

The reluctance to change the current legislative position has its explanations. The oppositions toward treatment refusal probably reflect the remnants of a communist philosophy in which the paternalistic state entirely controlled the life of the citizen. The individual was unable to make his own decisions, even on his or her own health. The communist regime even tried extensively to modulate or to efface religious feelings and beliefs, and among them those related to the afterlife and eternity; a wide anticlerical and antireligious campaign was promoted in 1967 with prolific discourses of the Albanian dictator [24]. The campaign culminated in a complete atheistic state for the following 25 years; obviously the overall oppression would have restricted the end-of-life decisions or the way they were legally and socially conceived. The atheistic state deformed the cultural and the social background, but the influence of communism and of the governing Party even in the '*way how life evolves*', to quote a Romanian author, was a much larger phenomenon, rather than being an Albanian peculiarity [25].

Strangely enough, the only Albanian Code that mentions and stipulates treatment refusal (as a notion) is the Naval Code. According to article 149 of this Code, the skipper is obliged to report every probable case of treatment refusal [26]. Compared to the overall hesitation concerning treatment refusal within Albanian territory, it seems that such 'slack' tolerance in the Naval Code may be demanded by the circumstances: other rules may apply when navigating in international waters, and when there may be passengers from other nationalities.

As said above, the role confusion and the lack of appropriate terminology in Albanian medical and legal settings are deleterious to the general conception of a 'death in dignity', and toward the application of advance directives for near-death and irreversible conditions. Such a terminological and legal vacuum has been felt elsewhere; in Italy for example, where during the last two decades the number of Albanian immigrants has considerably raised, bi- and multilingual brochures are made available from the social services. In an interesting part of a brochure produced from the social and medical services of the Italian region Emilia-Romagna (the brochure stamped in Albanian language under the title *Relationship with the elderly person*), an entire paragraph is dedicated to the 'meaning of the death'; all along the lines authors used synonymously the Albanian terms together with the Italian ones (for example, *vdekje - morte *[death]; *humbje - perdita *[loss]; *dinjitet - dignitá *[dignity]), thus underlining the potential conceptual discrepancies between the languages, and the cultures involved [27].

It seems that end-of-life decisions, treatment refusal and DNR requests in Albania are hazardous options. The rationale of their application is influenced by a mixture of religious beliefs, death coping-behaviors and above all, by an immense confusion concerning the role of the proxies as the decision-maker. Some of the options we suggest to improve the overall picture are through raising the public and professional awareness, through adopting more advanced experiences from other countries, and through discussing the issues in appropriate medical and legal forums.

Nevertheless, the Albanian tradition is familiar with the notion of 'amanet', a living will that predominantly deals the property and inheritance issues, but that in some cases will include advance directives regarding the last days of life, or near-death situations. If we have no doubts about the general notion of free will, like other authors who have raised suspicions about its existence [14], then there should be no obstacle to duly registering and honoring an advance directive, formulated by a competent person. The overall reticence, both culturally and legally imposed and sustained, regarding treatment refusal and end-of-life decisions in Albania has to be addressed by medical professionals, in the appropriate instances, and should be duly translated in legislative, regulatory and normative acts that will respond to the dilemmas described above.

## List of abbreviations

DNR: do-not-resuscitate; DNAR: do-not-attempt-resuscitation; ICU:intensive care unit; CPR: cardiopulmonary resuscitation; DOA: death-upon-arrival; MOLST: medical orders for life-sustaining treatment.

## Competing interests and funding

The authors have no competing interests to declare. No funds were granted to support the present paper.

## Authors' contributions

GV wrote the paper, revised it during the pre-publication process and compiled the literature. JK mentored the writing of the paper. Both authors read and approved the final manuscript.

## Pre-publication history

The pre-publication history for this paper can be accessed here:

http://www.biomedcentral.com/1472-6939/12/12/prepub
